# Editorial: Women in psychiatry 2024: addictive disorders

**DOI:** 10.3389/fpsyt.2025.1726772

**Published:** 2025-10-30

**Authors:** Stefania Chiappini, Annagiulia Di Trana

**Affiliations:** ^1^ UniCamillus University of Medical Sciences via di S. Alessandro, Rome, Italy; ^2^ National Center on Addiction and Doping, National Institute of Health, Rome, Italy

**Keywords:** women psychiatrists, addictive disorders, behavioural addiction, precision psychiatry, early identification

Addictive disorders continue to pose a major global public health challenge, affecting individuals, families, and communities across all demographics. Yet, addiction does not occur in a vacuum—it intersects with gender, social structures, mental health comorbidities, and stigma in ways that require a nuanced, multidisciplinary, and inclusive approach. Historically, addiction research, diagnosis, and treatment frameworks have been predominantly shaped by male-centric models, often overlooking the distinct biopsychosocial dimensions of addiction in women. Fortunately, this is changing.

This Research Topic aims to celebrate the achievements, perspectives, and contributions of women in the field of psychiatry, with a particular focus on the complex and evolving landscape of addictive disorders.

The original articles and reviews in this Research Topic reflect the growing body of work by women psychiatrists, psychologists and researchers who are not only advancing the science of addiction but are also reshaping the field through gender-sensitive frameworks, culturally informed interventions, and holistic care models.

Several themes appear throughout this Research Topic:

- The importance of early identification and intervention, especially in relation to the use and misuse of medications (Chiappini et al.) and craving for substances (Shmulevitz et al.).
- The role of co-occurring disorders—such as depression, anxiety, etc.—that often require integrated treatment approaches (Chiappini et al.).
- The growing significance of personalized and precision psychiatry in tailoring addiction treatments to meet the needs of diverse populations and the importance to inform patients regarding the treatments proposed (Heck et al.).
- The emergence of new types of behavioural addiction, such as problematic trading, and the acknowledgement of risk factors for its development (Loscalzo et al.).

In conclusion, this Research Topic offers new insights into the field of addictive disorders, significantly advancing knowledge and raising new fundamental questions that could serve as a fertile ground for further research.

